# Can GlideScope® videolaryngoscope be an alternative to direct laryngoscopy for child and infant tracheal intubation during chest compression?

**DOI:** 10.1007/s00431-015-2495-7

**Published:** 2015-01-31

**Authors:** Lukasz Szarpak, Lukasz Czyżewski, Andrzej Kurowski

**Affiliations:** 1 Department of Emergency Medicine, Medical University of Warsaw, Lindleya 4 Street, 02-005 Warsaw, Poland; 2 Department of Anesthesiology, Cardinal Wyszynski National Institute of Cardiology, Warsaw, Poland; 3 Department of Nephrologic Nursing, Medical University of Warsaw, Warsaw, Poland

In the article from Rodriguez-Nunez et al. entitled “Tracheal intubation of pediatric manikins during ongoing chest compressions. Does GlideScope® videolaryngoscope improve pediatric residents’ performance?” [1], the authors conducted a study on the time taken to intubate children and infants using the GlideScope and Miller (MIL) laryngoscopes. Due to the small number of participants in the aforementioned trial and a lack of data concerning the effectiveness of individual attempts at endotracheal intubation (ETI), decided decision was made to conduct extended research on intubation using the GlideScope and Miller Laryngoscopes.

This study was approved by the Program Committee of the International Institute of Rescue Research and Education (Approval 11.2014.03.23, November 3, 2014). This was a randomized non-blind crossover simulation trial with 112 paramedic participants; none of whom had prior experience with the GlideScope. In the child intubation scenario, we used a PediaSIM CPR training manikin (FCAE HealthCare, Sarasota, FL, USA), and a Lucas-2 device was used for chest compression. In the infant ETI scenario, we used a Laerdal®ALS Baby™ (Laredal, Norway) and chest compression was performed using the two-thumbs technique.

There was a statistically significant difference between the GlideScope and the MIL in the infant scenario in success of the first intubation attempt (96.5 vs. 60.7 %; *p* < 0.001), overall success rate (100 vs.83 %; *p* < 0.001), and time to successful intubation (34.6 vs.27.3 s; *p* = 0.023) (Table Supplementary data). In the child intubation scenario, there was a statistically significant difference in success of the first intubation attempt (100 vs.54.5 %; *p* < 0.001) and overall success rate (100 vs.82.1 %; *p* < 0.001); however, there were no significant differences in the time to successful intubation (36.6 vs.35.4 s; *p* = 0.085).

In summary, although the time of intubation using the GlideScope is higher in infant and child intubation scenarios, the higher effectiveness of the first intubation attempts and the higher overall effectiveness of intubation using the GlideScope suggest in favor of using videolaryngoscopy during child and infant intubation with chest compressions.

## Electronic supplementary material

Below is the link to the electronic supplementary material.

ESM 1 (DOCX 15 kb)
