# Supplementary material for: Can GlideScope® videolaryngoscope be an alternative to direct laryngoscopy for child and infant tracheal intubation during chest compression?
Source: Eur J Pediatr. 2015 Jan 31;174(7):981–2. doi: 10.1007/s00431-015-2495-7 (PMC4475241; doi:10.1007/s00431-015-2495-7)
Supplement: Supplementary file 1 — (DOCX 15 kb) [file 431_2015_2495_MOESM1_ESM.docx]

| Table. Time to and Success of Intubation. | | | | | | |
| --- | --- | --- | --- | --- | --- | --- |
| **Scenario** | **Intubation method** | **Time to intubation (s) [mean(SD)]** | **Tracheal intubation attempts** | | | |
|  |  |  | **First** | **Second** | **Third** | **Failed** |
| Infant | Miller | 27.3 (16.4) | 60.7% | 79.5% | 83.0% | 17.0% |
|  | GlideScope | 34.6 (5.6) | 96.4% | 100% | 100% | 0.0% |
| Child | Miller | 35.4 (13.5) | 54.5% | 77.7% | 82.1% | 17.9% |
|  | GlideScope | 36.6 (9.5) | 100% | 100% | 100% | 0.0% |
